# Microwave Cooking of Some or All High Starch Ingredients of Cattle Feed Concentrate Improves Nutritional Value and In Vitro Bioavailability

**DOI:** 10.3390/ani14203028

**Published:** 2024-10-19

**Authors:** Sukanya Poolthajit, Suriyanee Takaeh, Waraporn Hahor, Nutt Nuntapong, Wanwisa Ngampongsai, Karun Thongprajukaew

**Affiliations:** 1 Division of Health and Applied Sciences, Faculty of Science, Prince of Songkla University, Songkhla 90110, Thailand; sukanyapoolthajit@rmutl.ac.th (S.P.); suriyanee.ta@gmail.com (S.T.); waraporn.hahor@gmail.com (W.H.); 2 Kidchakan Supamattaya Aquatic Animal Health Research Center, Aquatic Science and Innovative Management Division, Faculty of Natural Resources, Prince of Songkla University, Songkhla 90110, Thailand; nutt.n@psu.ac.th; 3 Animal Production Innovation and Management Division, Faculty of Natural Resources, Prince of Songkla University, Songkhla 90110, Thailand; wanwisa.n@psu.ac.th

**Keywords:** carbohydrate, cattle feed, concentrate quality, microwave irradiation, pepsin–cellulase digestibility

## Abstract

Microwaving has been used to qualitatively improve feed ingredients prior to including them in cattle feed. However, it is not known whether feed ingredients should be microwaved separately or in a mixture before being included in cattle feed concentrates. In the current study, the effects of the partial and full microwaving of high starch ingredients were investigated regarding their impact on the nutritional composition, physicochemical properties, and in vitro digestibility of feed concentrates. The cassava was microwave-irradiated before being mixed with other ingredients (MC, 35% of formulation). A microwave-cooked cassava–corn meal mixture (MCC, 45% of formulation) and a combination of all solid components (MSI, 96% of formulation) were also compared. A feed containing non-microwaved ingredients was formulated and served as the control (NM). Significantly altered proximate compositions and nutritive profiles were observed in all the microwaved components (*p* < 0.05). The observed modifications in pHs, water absorption capacities, thermal properties (differential scanning calorimetry), diffraction patterns (X-ray diffractometry), and microstructures (scanning electron microscopy) indicated enhanced enzymatic hydrolysis in vitro. A higher cellulase organic matter solubility and digestible organic matter were observed in the MCC and MSIs feeds relative to the control NM (*p* < 0.05). These findings indicate a combination of components could be added to feedstock before it is microwave processed to potentially include this pretreatment in the feed production process.

## Simple Summary

Before adding feed ingredients to cattle feed, their quality can be improved by microwaving them. It is unknown, therefore, if feed components should be combined or microwaved separately before being added to concentrates for cattle feed. The current study examined the effects of partial and complete microwaving on the nutritional composition, physicochemical characteristics, and in vitro digestibility of high starch components. Cassava was microwave-irradiated prior to being mixed with additional components (35% of formulation). A blend of microwave-cooked cassava and corn meal (45% of formulation) and a combination of all solid components (96% of formulation) were also compared. A feed made only of non-microwaved components served as the control. Our findings indicate microwave processing of some or all of the solid starch ingredients improved the approximate chemical composition, physicochemical properties in aspects to enhance enzymatic digestion, and in vitro pepsin-cellulase digestibility of ruminant. However, pretreating cassava alone did not yield as good results as pretreating all solid components or cassava plus maize meal. These results suggest that a pretreatment might be included in the feed manufacturing process by adding a combination of ingredients to the feedstock prior to microwave processing.

## 1. Introduction

The correct feeding of cattle helps to maximize production. Farmers typically give cattle bulk feed containing more than 18% fiber, since cattle can extract energy from fibrous foods with the rich and diverse microbiota in their rumen [1]. The main energy source in beef cattle feed is usually starch, which is widely available [2]. In Thailand, the sources of starch for beef cattle feed include ground corn and cassava chips, which are typically used at high inclusion levels. Management systems often encourage the inclusion of high amounts of both starch sources to support growth performance and fat accumulation, but cassava and corn grain starch differ in terms of their solubility and fermentability in the rumen [3]. Kathrin et al. [4] found that an excess of ruminal non-degradable starch in the diet resulted in a low efficiency for ruminal nitrogen utilization and microbial protein synthesis and also decreased starch utilization efficiency. Thus, many researchers have attempted to elucidate an appropriate method to modulate the degradability of starch sources in the rumen by enhancing feed efficiency through altering the nature and amount of starch available to rumen microbes. This is because, in the aforementioned study, the rumen fermented more than 70% of the starch that was ingested, with the remaining fraction either leaving through digestion in the small intestine or fermentation in the large intestine [5,6].

The quality of animal feedstuffs can be improved by pre-cooking. In general, microwave processing has achieved general acceptance in the food industry and among chemical engineers [7]. Microwave processing heats materials throughout, producing faster heating rates and shorter processing times than conventional heating processes [8]. In feed production, microwave processing can improve feedstuff utilization by altering some of the physicochemical properties that enhance enzymatic hydrolysis [9,10]. However, the effects of partial and full microwave pretreatment on the bioavailability of feed and the differences between them are not known and need to be assessed.

When feedstuffs that contain high amounts of carbohydrates are pretreated by microwave processing, starch gelatinization can occur [11,12]. However, unpurified raw ingredients also contain a protein fraction, so the pretreatment inevitably denatures the protein as well. Generally, such changes in pretreated feed components affect the physicochemical properties that enhance enzymatic hydrolysis in vitro, such as pH, turbidity, water absorption capacity, relative crystallinity, and microstructure [9,10,13] and can be evaluated by analyzing these properties.

It is possible that a partial or complete processing of raw materials, particularly using microwaves, could improve the nutritional composition and utilization of foods. Therefore, the objective of the present study was to compare the effects of the partial versus full microwaving of ingredients on the chemical composition, physicochemical properties, and in vitro pepsin–cellulase digestibility of feed concentrates. Findings from the current study can provide a practical production guideline for cattle feed concentrates.

## 2. Materials and Methods

### 2.1. Pretreatment of Ingredients and Production of Experimental Feed Concentrates

Feed ingredients were purchased from local merchants in the Songkhla Province, Thailand. The experimental feed concentrates were prepared according to a formulation for Thai native cattle reported by Poolthajit et al. [14] (Table 1).

The ingredient sources in the formulation included cassava chips, corn meal, rice bran, palm kernel cake, dried cassava pulp, and soybean meal. The control feed concentrate (NM) included all the components of the reported formulation but none of the components were pretreated by microwave heating. Three experimental feed concentrates were then formulated that included all the components of the NM. However, in these feed concentrates, different starch sources were processed by microwave cooking. In the first (MC), the cassava chips were processed; in the second (MCC), the cassava chips and corn meal were processed and in the third (MSIs), all six starch sources were processed. Based on our assumption, the proportion of starchy ingredients will be gradually gelatinized by the microwaving process, which will affect the chemical composition, the physicochemical properties, and enzymatic digestion in vitro of the prepared feed concentrate. To process the starch sources, the ingredients were finely ground and sieved through a 1 mm mesh. The ground components were combined by weight according to the proportions in Table 1. For preparing the microwaved ingredients, 300 g of feedstuffs was mixed with distilled water (2:1 *w*/*v*) in a round microwave-safe plastic container (23 cm diameter × 10.5 cm height), and then irradiated in a microwave oven (MW71B; Samsung, Kuala Lumpur, Malaysia) at 700 W for 11 min [15]. After microwave processing, the oven-dried MC, MCC, and MSIs were mixed with the other components of the formulation. Urea, sulfur, salt, dicalcium phosphate, and a premix were included and untreated in all formulations. The obtained feed concentrates were kept at 4 °C until used.

### 2.2. Preparation of Feed Samples for Chemical Analysis

The concentrates were ground, sieved, and freeze-dried (Delta 2-24 LSC; Martin Christ Gefriertrocknungsanlagen GmbH, Osterode am Harz, Germany) for 24 h to eliminate excess moisture. The dried samples were kept in a desiccator for a later analysis of their proximate chemical composition, physicochemical properties and in vitro digestibility.

### 2.3. Proximate Chemical Compositions and Nutritive Profiles

The AOAC [16] standard method was used to determine the proximate chemical compositions of the prepared samples. Contents of the dry matter (DM, No. 967.03), ash (No. 492.05), crude protein (CP, No. 984.13), and ether extract (EE, No. 920.39) were determined. The method reported by Van Soest et al. [17] was used to analyze the neutral detergent fiber (NDF) and acid detergent fiber (ADF) contents. The formula for calculating a non-fiber carbohydrate (NFC, %) was 100 − (% CP + % EE + % NDF + % ash). An adiabatic bomb calorimeter (AC500; LECO Corporation, St. Joseph, MI, USA) was used to calculate gross energy (GE).

Fourier transform infrared spectroscopy (FTIR; FW-4; Thermo Fisher Scientific, Waltham, MA, USA) was applied to assess qualitative changes in the nutritive values of the experimental feed concentrates. A total of 2 mg of the sample was combined with 200 mg KBr in a vacuum dryer for 24 h. Using an infrared tablet press, the mixture was uniformly ground and pressed into a translucent tablet of 0.25 mM. The spectra were acquired in the mid IR range (4000 to 400 cm^−1^) at a resolution of 4 cm^−1^. As a control, a KBr pellet without a sample was scanned in identical conditions. The infrared spectra were processed using OMNIC 8.0 software (Nicolet Analytical Instruments, Madison, WI, USA), and after a baseline correction of the original map, the sample and control spectra were compared.

### 2.4. Physicochemical Properties

#### 2.4.1. pH

The dried feed concentrates were combined with 6.25 mL of distilled water at room temperature. The mixture was then stirred for 10 min, as described by Sokhey and Chinnaswamy [18]. Suspension pH was measured with a pH meter (Five Easy F20; Mettler-Toledo GmbH, Greifensee, Switzerland).

#### 2.4.2. Water Solubility

Water solubility was ascertained using the method of Chung et al. [19]. In summary, 1 g of sample was combined with 10 mL of water, gently mixed at ambient temperature for 1 h, then centrifuged at 1500× *g* for 10 min. After collection, the supernatant was dried for 48 h at 60 °C and weighed. The weight of the dissolved particles in the supernatant was divided by the weight of the dry solids in the original sample to determine the water solubility of the sample.

#### 2.4.3. Water Absorption Capacity (WAC)

Water absorption capacity was determined as described by Jitngarmkusol et al. [20]. In brief, 5 mL of distilled water was combined with 2 g of dried feed sample, left to stand at room temperature for 30 min, and then centrifuged at 2000× *g* for 10 min. The sample was reweighed after the supernatant was decanted, and the WACs were reported as grams of water absorbed per gram of dry sample.

#### 2.4.4. Differential Scanning Calorimetry (DSC)

The thermal transition properties of the feed concentrate samples, including the onset (T_o_), peak (T_p_), and conclusion (T_c_) temperatures, temperature range (T_c_–T_o_), and transition enthalpy (∆H), were determined by DSC (DSC7; Perkin Elmer, Waltham, MA, USA). An aluminum pan containing 3 mg of a sample was sealed, left to acclimate at ambient temperature for 1 h, and then heated from 40 to 400 °C at 10 °C min^–1^.

#### 2.4.5. Diffraction Patterns and Relative Crystallinity (RC)

The diffraction patterns and RC of the feed samples were determined by X-ray diffractometry (XRD, X′ Pert MPD, Philips, Amsterdam, The Netherlands) at 40 kV and 30 mA. The diffraction angles were measured between 4 and 90° (2θ), but only reported between 4 and 40°. Relative crystallinity was assessed from the area under each notable peak and the total area under the curve.

#### 2.4.6. Microstructure and Topography

The microstructure of the feed samples was observed with scanning electron microscopy (SEM, Quanta 400; FEI, Brno, Czech Republic). The dried samples were fastened to aluminum stubs with double-sided sticky tape and coated with gold. Scanning electron microscopy was operated at an acceleration voltage of 20 kV. Surface microstructures were captured at 250- and 1500-times magnification. The high-resolution SEM images were preprocessed for background subtraction and flattening in Gwyddion 2.65, a free, open-source program, before being imported into three-dimensional topographic studies. Using surface analysis tools to measure roughness, the arithmetic average roughness, the maximum height of the roughness, and the maximum roughness valley depth were computed.

### 2.5. In Vitro Pepsin–Cellulase Digestibility Screening

The digestibility of the feed concentrate samples was assessed using the pepsin–cellulase technique according to De Boever et al. [21]. In brief, 300 mg of a sample was placed in a glass filter crucible, and 30 mL of a pepsin-hydrochloric acid solution (2 g of pepsin in 1 L of 0.1 M HCl) was added. The crucible was incubated at 40 °C for 24 h and shaken every 5 h. The crucible was then submerged in a water bath at 80 °C for 45 min. Afterward, the residue was dissolved in distilled water and the solution was removed. After adding a cellulase buffer (Onozuka R-10^®^; SERVA Electrophoresis GmbH, Heidelberg, Germany) to 30 mL of the solution at 40 °C, the mixture was incubated for 24 h and shaken every 5 h within this timeframe. At 40 °C, the solution was aspirated, and the leftover material was washed with distilled water. In order to calculate the dry matter value, the digested portion was dried at 103 °C, and the residue was burned at 550 °C to estimate the cellulase organic matter solubility (COMS), which was computed as follows: COMS (%) = (W_o_ − W_t_/W_o_) × 100, where W_o_ and W_t_ represent the weights of the organic matter (OM) prior to and following incubation. Following De Boever et al. [21], the digestible organic matter (DOM) and metabolizable energy (ME) of the feed samples were computed as follows: DOM (%) = (0.973 × COMS) − 2.49, and ME (MJ kg^–1^ DM) = (0.150 × COMS) + (0.241 × EE) − 0.99.

### 2.6. Statistical Analysis

The trials were conducted using a completely randomized design. The target power test of 0.8 was utilized to determine the minimal sample size using R 3.6.0. Data were presented as means ± SEM. The Statistical Package for Social Sciences Version 22 (SPSS Inc., Chicago, IL, USA) was used to calculate all of the statistical values. The percentages were transformed using the arc sine function before an analysis. The normality and homogeneity of variance were examined. Duncan’s multiple range test was used as a post hoc test in a one-way analysis of variance to assess the differences in means across the treatment groups. If the *p*-value was less than 0.05, the hypothesis was rejected.

## 3. Results

### 3.1. Chemical Compositions and Nutritive Profiles of Concentrates

The chemical compositions of the experimental feed concentrates are shown in Table 2.

Microwave processing had significant effects on the DM (*p* < 0.001), OM (*p* = 0.001), CP (*p* = 0.043), EE (*p* = 0.034), NDF (*p* = 0.006), and ash (*p* = 0.001) contents, while the ADF (*p* = 0.051), NFC (*p* = 0.086), and GE (*p* = 0.052) were unchanged. The dry matter contents were significantly higher in the MCC and MSIs concentrates relative to the NM. The last treatment, in which all solid ingredients were processed together, had a lower OM content compared to the MC or MCC, a higher CP content, and a significantly lower EE content. The neutral detergent fiber and ash contents were lower in all the processed feed concentrates relative to the NM.

**Figure 1:**
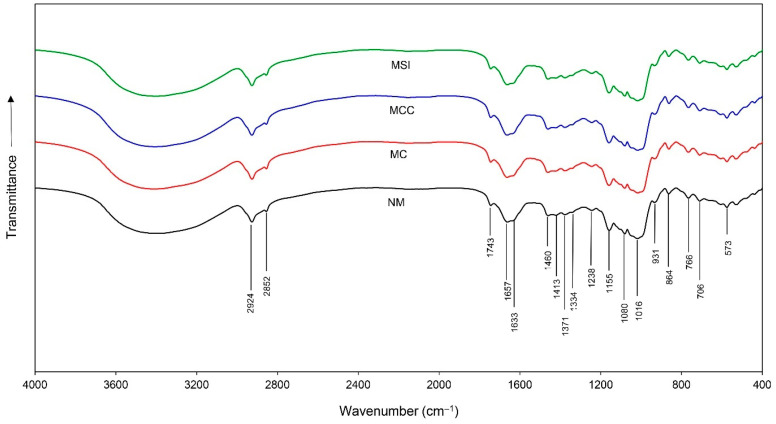
FTIR spectra of cattle feed concentrates containing different grade levels of microwaved ingredients. A feed containing non-microwaved ingredients served as the control (NM) while feed concentrates containing microwaved cassava (MC), microwaved cassava and corn meal (MCC), or microwaved solid ingredients (MSIs) served as the experimental groups.

Though there were variations in the peak heights and intensities, overall, the FTIR spectra of all the formulations were similar. Within the range of 4000 to 400 cm^–1^, at least eighteen bands (2924, 2852, 1743, 1657, 1633, 1460, 1413, 1371, 1334, 1238, 1155, 1080, 1016, 931, 864, 766, 706, and 573 cm^–1^) were observed (Figure 1), which indicated the qualitative variations in the nutritional profiles, specifically in proteins, lipids, carbohydrates, and inorganic matter. The assignments of these bands are listed in Table 3.

### 3.2. Physicochemical Properties of Processed Concentrates

#### 3.2.1. pH, Water Solubility, and WAC

**Figure 2:**
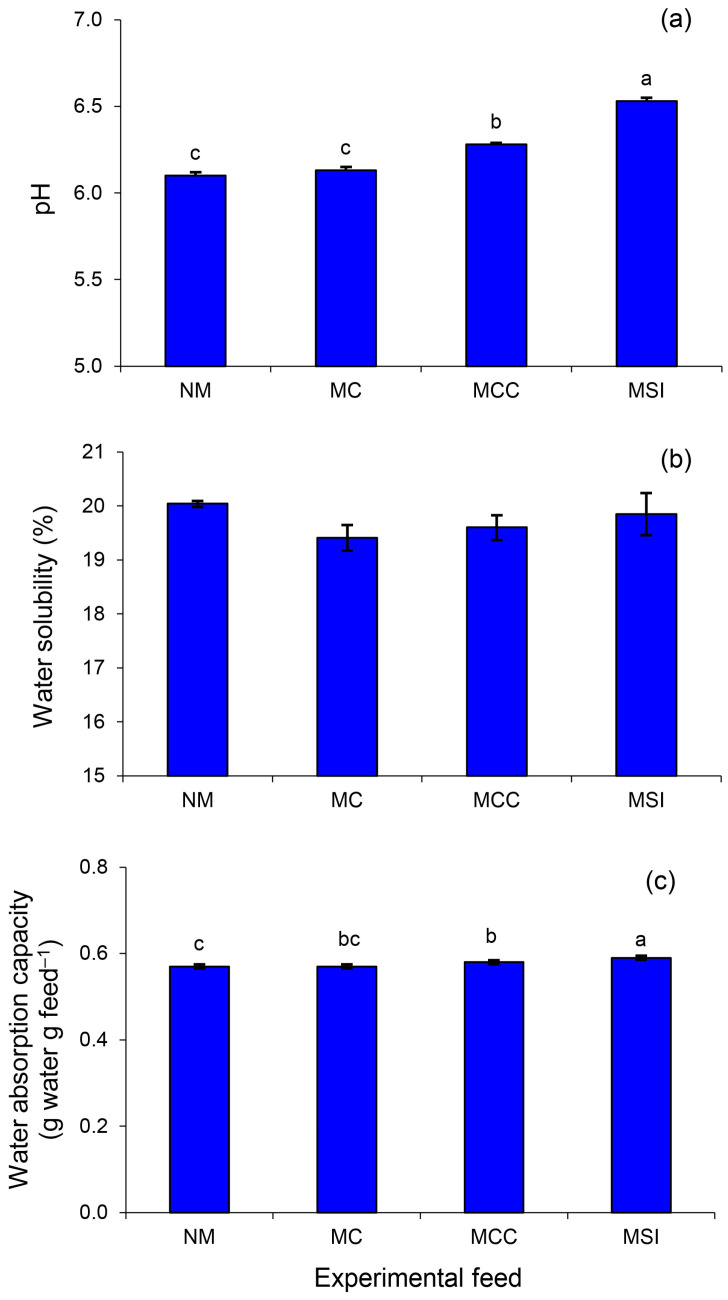
The pH (**a**), water solubility (**b**), and water absorption capacity (**c**) of cattle feed concentrates containing different grade levels of microwaved ingredients. A feed containing non-microwaved ingredients served as the control (NM) while feed concentrates containing microwaved cassava (MC), microwaved cassava and corn meal (MCC), or microwaved solid ingredients (MSIs) served as the experimental groups. Data are expressed as means ± SEM (*n* = 3). Significant differences between treatments are indicated by different superscripts (*p* < 0.05).

The pH of the feed concentrates was significantly altered by the microwave pretreatments. When more components were added, the pH levels increased (*p* < 0.001, Figure 2a). No differences in water solubility were observed between the non-processed and processed feed concentrates (*p* = 0.063, Figure 2b). The water absorption capacity values were significantly influenced by the addition of ingredients to the processed concentrate (Figure 2c). The highest WAC was observed in the MSIs concentrate followed by the MCC and MC.

#### 3.2.2. Thermal Transition Properties

The thermal characteristics of the feed concentrates are shown in Table 4.

Within the studied temperature range, one transition peak was observed, spanning a temperature range from 53.7 to 140 °C. Compared to the NM concentrate, the MCC and MSIs had higher T_o_, T_p_, T_c_, and T_c_–T_o_ while MC generally maintained these characteristics, except for T_c_–T_o_, which was lower than control. All the processed feed concentrates showed significantly lower ∆H than control; the MSIs had the lowest value while the MC and MCC were intermediate.

#### 3.2.3. Diffraction Patterns

**Figure 3:**
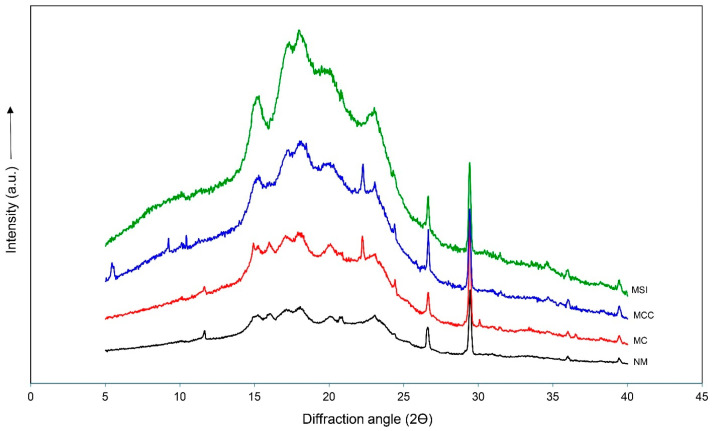
The diffraction patterns of cattle feed concentrates containing different grade levels of microwaved ingredients. A feed concentrate containing non-microwaved ingredients served as the control (NM) while feed concentrates containing microwaved cassava (MC), microwaved cassava and corn meal (MCC), or microwaved solid ingredients (MSIs) served as the experimental groups.

The four feed concentrates produced similar main peaks in their diffraction patterns (15.4, 18.3, 23.1, 26.7, and 29.4°) (Figure 3). However, small differences in the peaks were observed at diffraction angles of 15.6 to 21.5° and 23.7 to 30.0° (2θ). The relative crystallinity was unaffected by the pretreatment, giving values from 26.9 to 28.7%.

#### 3.2.4. Microstructure and Topography

**Figure 4:**
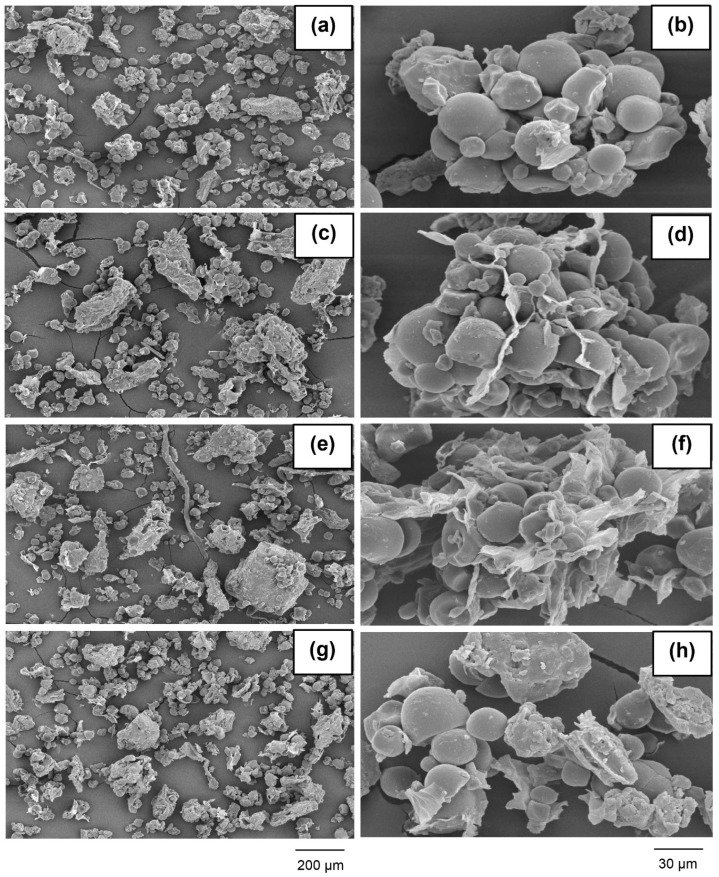
The microstructures of cattle feed concentrates containing different grade levels of microwaved ingredients. A feed containing non-microwaved ingredients served as the control (NM, **a**,**b**) while feed concentrates containing microwaved cassava (MC, **c**,**d**), microwaved cassava and corn meal (MCC, **e**,**f**), or microwaved solid ingredients (MSIs, **g**,**h**) served as the experimental groups. The micrographs were recorded at magnifications of 250× (**left**) and 1500× (**right**).

The microstructure of all four feed concentrates varied somewhat (Figure 4).

**Figure 5:**
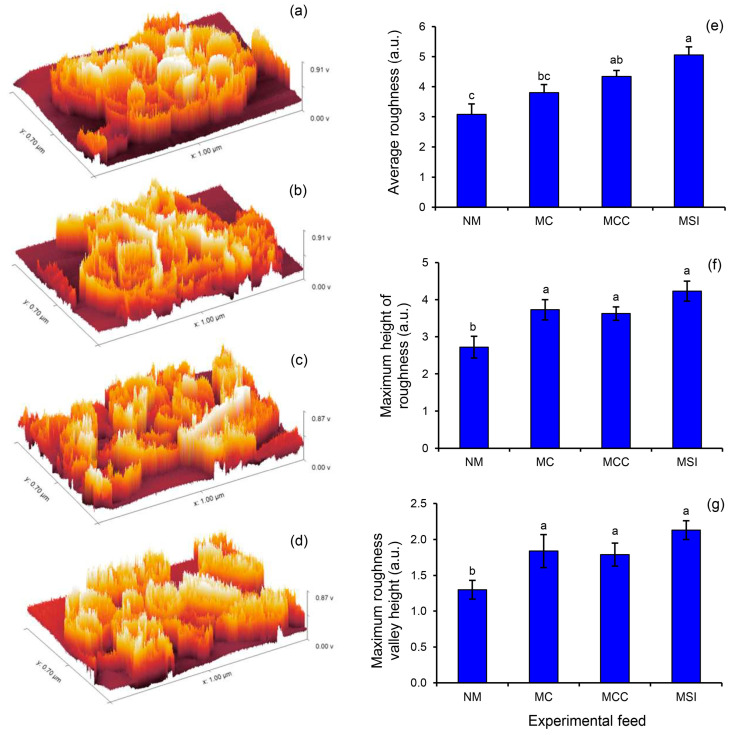
The three-dimensional topography of the cattle feed concentrates containing different grade levels of microwaved ingredients. A feed concentrate containing non-microwaved ingredients served as the control (NM, **a**) while a feed concentrate containing microwaved cassava (MC, **b**), microwaved cassava and corn meal (MCC, **c**), or microwaved solid ingredients (MSIs, **d**) served as the experimental groups. The topographic parameters were the arithmetic average roughness (**e**), maximum height of roughness (**f**), and maximum roughness valley height (**g**). Data are expressed as means ± SEM (*n* = 3). Significant differences between treatments are indicated by different superscripts (*p* < 0.05).

At a low magnification, all samples exhibited the same overall morphology of agglomerated irregular particles. At a high magnification, smooth surfaces and obvious swelling were observed. Granules of starch were connected and agglomerated in the MC and MCC. At a lower magnification, the MSIs feed concentrate presented certain irregular particles and irregularly scattered particles. The three-dimensional surfaces of the feed concentrates are illustrated in Figure 5. The arithmetic average roughness, the maximum height of roughness, and the maximum roughness valley height were greater in all the processed feed concentrates relative to the NM (*p* = 0.010). The MSIs showed the highest values.

### 3.3. In Vitro Digestibility

The digestibility of the COMS, DOM, and ME are shown in Table 5.

The highest COMS (*p* = 0.036) and DOM (*p* = 0.036) were observed in the MCC followed by the MSIs and MC. No significant differences in the ME were observed across the four feed concentrates (*p* = 0.757).

## 4. Discussion

The proximate chemical composition and FTIR spectral analysis indicated significant changes in the nutritional profiles across the four feed concentrates. The MSIs concentrate showed a significantly increased CP content while the MC and MCC did not. The higher parts of the molecular weight aggregates may result from the processing of mixed solid materials, which may cause covalent cross-linkages of proteins and other N-containing chemicals [31]. A microwave pretreatment can cause significant changes in some proximate compositions, depending on the type of feedstuff or the proportion of ingredients that have been pretreated. Nitrogen availability can be increased by physical changes such as unfolding or changes in protein composition brought on by C-N bond scissions in the polypeptide chains backbones [15]. In this study, these changes resulted in an increase in CP content when analyzed by the Kjeldahl method.

The EE levels of the MSIs feed concentrate were reduced because the fats were rapidly oxidized and degraded by microwave irradiation [32]. However, the EE content of the MC and MCC groups were not different from the control group, which used the same types and proportions of ingredients. These data suggest that the interaction caused by microwave exposure in some or all of the starch groups affected the EE changes. For the indigestible elements, all the microwaved groups showed variations in NDF content (which includes cellulose, hemicellulose, and lignin), but not in ADF content, which also contains cellulose and lignin. This result suggests that the microwave processing significantly reduced the amount of hemicellulose. Generally, this treatment breaks the biomass down into smaller pieces and makes it expand, which breaks down the lignin and hemicellulose [33].

Compared to the control, all three processed feed concentrates showed a decrease in ash content, with the degree of ash loss being directly related to the processing. The dissolution of ingredients before the microwaving and drying process after pretreatment may reduce the amount of minerals available. Takaeh et al. [10], in contrast, reported increased ash contents in microwave-irradiated cassava chips relative to the native form. These conflicting results suggest that complex interactions between the substances take place during the microwave processing of feed. Alterations in the detailed compositions of nutrients caused significant changes in the amounts of OM and DM, without any effects on the amounts of NFC and GE.

The benefits of microwave feed processing extend beyond an improved chemical composition. The physicochemical characteristics of the feed are enhanced which facilitates enzymatic hydrolysis. All the processed feed concentrates showed significant increases in pH and WAC, with the MSIs concentrate exhibiting the greatest values. While a higher WAC is correlated with the fiber content that interacts with water [34], an increased pH may be the result of the hydroxyl groups being released during lignocellulosic breakdown [35].

When starch is cooked with the water present, the granules expand, and the crystalline structure of the starch breaks down to create amorphous areas. When the amorphous regions absorb water and swell to a gel phase, a semi-cooperative process known as gelatinization takes place, putting a strain on the crystalline regions. According to Wang et al. [36], this process strains the crystallites, causing them to cooperatively melt at a lower temperature than those that are not connected to the gel phase. This molecular disordering is often observed as an endothermic phenomenon and can be quantified using DSC. The thermal properties of the MCC and MSIs concentrates in this investigation demonstrated increasing trends. The creation of complexes between amylose and other chemicals, as well as the amylose–amylose interactions or the amylose–amylopectin chain interactions, could be the cause of the elevated temperatures [37]. After microwave processing, cleaved polymers were observed as a result of the large changes in the T_c_–T_o_ ranges produced by the shifts in the onset and conclusion points [38]. The MCC and MSIs feed concentrates exhibited a relatively wide T_c_–T_o_ range, indicating the substantial heterogeneity of the cleaved polymers provided by the microwaving process. On the other hand, the relatively low ∆H indicates the amount of energy required to gelatinize the native form [38]. These temperature responses showed that the overall quality of the feed concentrates, especially MCC and MSIs, were significantly improved by microwave heating.

The crystal structure of the feed concentrates was determined by the XRD analysis. There were strong peaks in the diffraction patterns at 15.4, 18.3, 23.1, 26.7, and 29.4° (2θ). This result was in line with the principal ingredient in the concentrates, cassava chips, which produce strong peaks at 15.1, 17.1, 18.0, and 26.6° [10]. These peaks correlate to the C-type mixed crystal structure [39]. While the microwave treatment changed the XRD readings for certain raw materials significantly [40,41,42], our results showed that microwave irradiation had no effect on the RC values. Nevertheless, it is evident from the XRD data that the partial and complete microwaving of solid ingredients has a direct effect on the quality of the resulting feed concentrate.

The processed feed concentrates had rough surfaces with agglomerations that included clear film, whereas the NM had smooth surfaces with evident swelling. These features are indicative of the gelatinization of starch. However, the concentrates did not consist solely of starch, but also contained other nutrients that also affected the surface structure. Based on topographic investigations, three of our observed parameters indicated that the surface roughness of the processed feed concentrates was increased relative to the NM. This feature relates to the enzymatic hydrolysis that occurs throughout the digestive system of cattle. The surface roughness improves the efficiency of enzymatic hydrolysis by increasing the surface-to-volume ratio and allowing a large enzyme loading [35]. Thus, it is possible that cooking the mixture in a microwave might be more advantageous for the animals.

Fungal cellulases have been used in an enzymatic approach that simulates ruminal breakdown to determine the bioavailability of feed concentrate. In the current investigation, we found that the digestibility of DOM and COMS was highest in the MCC feed concentrate, with MSIs coming in second. This study found that adding other starch components to cassava chips increased the digestibility of the feed concentrate. Our findings support the findings of Hahor et al. [9], who reported that the in vitro digestibility of aquafeed was improved more by microwave processing only the carbohydrate components than by processing all the solid ingredients of the formulation. The complex interplay of the components seems to play a major role in modifying feed bioavailability in vitro and improving the chemical composition and physical characteristics in ways that facilitate enzymatic hydrolysis. The specific determination of the liberated sugars, such as maltose or glucose, after determining the in vitro digestibility of the feed concentrate is needed for a better understanding of carbohydrate digestion in cattle.

## 5. Conclusions

A microwave pretreatment for some or all of the solid starch ingredients in cattle feed concentrate mostly improved the proximate chemical composition, physicochemical characteristics, and in vitro pepsin–cellulase digestibility of the concentrate. However, pretreating the cassava and corn meal (45% of the feed concentrate formulation) or all the solid ingredients (96% of the feed formulation) provided more favorable results than pretreating the cassava alone (35% of the feed formulation). Even though some of the commercially available feedstuffs may have already been thoroughly denatured and precooked during the production process, the bioavailability of the prepared feed was effectively improved by combining and microwaving them again. It might be feasible to incorporate this pretreatment into the feed production process by adding a mixture of components to feedstuff before microwave processing all the components together. However, this study did not specifically look at starch digestibility, so it may not be completely clear how much it enhances starch use. In addition, the economic cost of producing feed using this method should be studied before it is actually used. Other methods of hydrothermal treatments, such as boiling, extruding, and steaming, can also be compared to microwaves.

## Figures and Tables

**Table 1:** The formulations of experimental cattle feed concentrates containing different grade levels of microwaved ingredients. The addition of microwaved ingredients in the formulation is marked by ‘+’ while the utilization of untreated ingredients is marked by ‘−’.

Ingredient	Inclusion (% of Dry Matter)	Experimental Feed			
		NM	MC	MCC	MSI
Cassava chip	35	−	+	+	+
Corn meal	10	−	−	+	+
Rice bran	10	−	−	−	+
Palm kernel cake	19	−	−	−	+
Dried cassava pulp	14	−	−	−	+
Soybean meal	8	−	−	−	+
Urea	1.8	−	−	−	−
Sulfur	0.2	−	−	−	−
Salt	0.5	−	−	−	−
Dicalcium phosphate	1.0	−	−	−	−
Premix ^1^	0.5	−	−	−	−

A feed concentrate containing non-microwaved ingredients served as the control (NM) while feed concentrates containing microwaved cassava (MC), microwaved cassava–corn meal (MCC), or microwaved solid ingredients (MSIs) served as the experimental groups; ^1^ The premix (per 1 kg) contained vitamin A: 10,000,000 IU; vitamin E: 70,000 IU; vitamin D: 1,600,000 IU; Fe: 50 g; Zn: 40 g; Mn: 40 g; Co: 0.1 g; Cu: 10 g; Se: 0.1 g; and I: 0.5 g.

**Table 2:** The proximate chemical compositions (based on dry matter) of the experimental cattle feed concentrates containing different grade levels of microwaved ingredients.

Chemical Composition	Experimental Feed			
	NM	MC	MCC	MSI
Dry matter (%)	96.5 ± 0.1 ^b^	96.7 ± 0.1 ^b^	97.2 ± 0.1 ^a^	97.3 ± 0.1 ^a^
Organic matter (%)	94.0 ± 0.1 ^d^	94.6 ± 0.1 ^b^	94.8 ± 0.1 ^a^	94.3 ± 0.1 ^c^
Crude protein (%)	14.6 ± 0.4 ^b^	13.3 ± 0.1 ^b^	15.7 ± 1.5 ^ab^	18.1 ± 0.7 ^a^
Ether extracts (%)	3.71 ± 0.01 ^a^	3.47 ± 0.14 ^a^	3.01 ± 0.25 ^ab^	2.69 ± 0.27 ^b^
Neutral detergent fiber (%)	26.4 ± 0.3 ^a^	25.4 ± 0.1 ^b^	24.1 ± 0.3 ^c^	24.7 ± 0.2 ^bc^
Acid detergent fiber (%)	21.2 ± 0.4	20.6 ± 0.4	20.5 ± 0.4	21.0 ± 0.3
Ash (%)	5.98 ± 0.10 ^a^	5.45 ± 0.01 ^c^	5.17 ± 0.01 ^d^	5.71 ± 0.02 ^b^
Non-fiber carbohydrate (%)	49.3 ± 0.1	52.4 ± 0.1	52.0 ± 2.1	48.8 ± 1.1
Gross energy (kcal kg^–1^)	4122 ± 2	4115 ± 3	4107 ± 9	4114 ± 4

A feed concentrate containing non-microwaved ingredients served as the control (NM) while feed concentrates containing microwaved cassava (MC), microwaved cassava–corn meal (MCC), or microwaved solid ingredients (MSIs) served as the experimental groups; ^a–d^ means in the same row with different superscripts indicate significant differences (*p* < 0.05).

**Table 3:** Tentative assignments of FTIR spectral peaks found in experimental feed concentrates containing different grade levels of microwaved ingredients.

Wavenumber (cm^–1^)	Tentative Band Assignment	Macromolecule	References
2924	*v*_as_ (CH_2_) stretching of methylene	Lipid	[22,23]
2852	*v*_s_ (CH_2_) stretching of methylene	Lipid	[22,23]
1743	*v* (C=O) stretching of esters	Lipid	[24]
1657	*v*_s_ (C=O) stretching of amide I	Protein	[25]
1633	*v*_s_ (C=O) stretching of amide I	Protein	[25]
1460	δ_as_ (CH_2_) bending of methyl	Lipid	[22,23]
	δ_as_ (CH_3_) bending of methyl	Protein	[24]
1413	δ_as_ (CH_2_) bending of methyl	Lipid	[22,23]
	δ_as_ (CH_3_) bending of methyl	Protein	[24]
1371	*v*_s_ (COO-) stretching of amino acid salt	Protein	[26]
	*v*_s_ (C=O) stretching vibrations of carboxylate	Carbohydrate	[27]
1334	*v*_s_ (C=O) stretching vibrations of carboxylate	Carbohydrate	[27]
1238	N (C=O) stretching and (C-OH) bending of deprotonated amino acid	Protein	[28]
1155	*v* (C-O-C) stretching of polysaccharide	Carbohydrate	[29]
1080	*v* (C-O-C) stretching of polysaccharide	Carbohydrate	[29]
1016	*v* (C-O-C) stretching of polysaccharide	Carbohydrate	[29]
931	C=C bending of alkene	Alkene	[26]
864	*v*_as_ (PO_4_^3−^) P-O asymmetric stretching of lipids	Lipid	[22]
766	δ (CO_3_^2−^) Out of O-C=O bending of oxalate	Inorganic	[24]
706	(CH^2−^), C-H rocking of lipids	Lipid	[22]
573	C-Br stretching of halo compound	Inorganic	[30]

**Table 4:** Thermal transition properties of experimental feed concentrates containing different grade levels of microwaved ingredients.

Thermal Parameter	Experimental Feed			
	NM	MC	MCC	MSI
T_o_ (°C)	53.7 ± 1.9 ^c^	53.9 ± 0.1 ^bc^	55.1 ± 0.7 ^a^	55.0 ± 0.2 ^ab^
T_p_ (°C)	86.3 ± 0.8 ^b^	86.7 ± 1.4 ^b^	91.4 ± 1.6 ^a^	91.7 ± 1.4 ^a^
T_c_ (°C)	130 ± 2 ^b^	127 ± 1 ^b^	137 ± 2 ^a^	140 ± 1 ^a^
T_c_–T_o_ (°C)	79.2 ± 2.0 ^b^	72.9 ± 0.4 ^c^	81.8 ± 2.4 ^ab^	84.8 ± 1.2 ^a^
∆H (J g^–1^)	94.2 ± 5.1 ^a^	81.4 ± 5.4 ^ab^	89.6 ± 3.2 ^ab^	75.4 ± 2.3 ^b^

A feed concentrate containing non-microwaved ingredients served as the control (NM) while feed concentrates containing microwaved cassava (MC), microwaved cassava–corn meal (MCC), or microwaved solid ingredients (MSIs) served as the experimental groups; T_o_, onset temperature; T_p_, peak temperature; T_c_, conclusion temperature; T_c_–T_o_, temperature range; ∆H, enthalpy; and ^a–c^ means in the same row with different superscripts indicate significant differences (*p* < 0.05).

**Table 5:** The pepsin–cellulase digestibility (based on dry matter) of experimental feed concentrates containing different grade levels of microwaved ingredients.

Digestibility	Experimental Feed			
	NM	MC	MCC	MSI
COMS (%)	82.3 ± 0.2 ^b^	82.9 ± 0.3 ^b^	84.6 ± 0.1 ^a^	83.9 ± 0.7 ^ab^
DOM (%)	77.6 ± 0.2 ^b^	78.1 ± 0.3 ^b^	79.8 ± 0.1 ^a^	79.2 ± 0.7 ^ab^
ME (MJ kg^–1^)	12.2 ± 0.1	12.2 ± 0.1	12.3 ± 0.1	12.2 ± 0.2

A feed concentrate containing non-microwaved ingredients served as the control (NM) while feed concentrates containing microwaved cassava (MC), microwaved cassava–corn meal (MCC), or microwaved solid ingredients (MSIs) served as the experimental groups; COMS, cellulase organic matter solubility; DOM, digestible organic matter; ME, metabolizable energy; and ^a,b^ means in the same row with different superscripts indicate significant differences (*p* < 0.05).

## Data Availability

The original contributions presented in the study are included in the article and Supplementary Materials, further inquiries can be directed to the corresponding author.

## Supplementary Materials

The following supporting information can be downloaded at: https://www.mdpi.com/article/10.3390/ani14203028/s1.
