# Supplementary material for: Microwave Cooking of Some or All High Starch Ingredients of Cattle Feed Concentrate Improves Nutritional Value and In Vitro Bioavailability
Source: Animals (Basel). 2024 Oct 19;14(20):3028. doi: 10.3390/ani14203028 (PMC11505226; doi:10.3390/ani14203028)
Supplement: Supplementary file 1 [file animals-14-03028-s001.zip › animals-3239603-supplementary.pdf]

	Treatment	Rep		ΔH (Jg^-1) Peak1	To (◦C) Peak1	Tp (◦C) Peak1
Beef	control (NM)		1	143.490	47.910	85.170
			2	100.450	53.950	87.830
			3	87.910	53.290	85.830
		mean		110.617	51.717	86.277
		SD		29.151	3.313	1.385
		SEM		16.831	1.913	0.800
	microwaved cassava (MC)		1	74.630	53.960	84.330
			2	92.060	54.450	89.170
			3	77.510	54.210	86.500
		mean		81.400	54.207	86.667
		SD		9.343	0.245	2.424
		SEM		5.394	0.141	1.400
	microwaved cassava-corn meal (MCC)		1	91.890	53.260	90.830
			2	93.570	55.330	94.500
			3	83.460	55.600	89.000
		mean		89.640	54.730	91.443
		SD		5.418	1.280	2.801
		SEM		3.128	0.739	1.617
	microwaved solid ingredients (MSI)		1	72.930	54.370	90.830
			2	79.910	54.910	94.500
			3	73.350	55.010	89.830
		mean		75.397	54.763	91.720
		SD		3.914	0.344	2.459
		SEM		2.260	0.199	1.420

Tc (◦C) Peak1	Tc–To (◦C) Peak1
130.150	82.240
133.990	80.040
128.590	75.300
130.910	79.193
2.779	3.547
1.604	2.048
126.970	73.010
126.580	72.130
127.710	73.500
127.087	72.880
0.574	0.694
0.331	0.401
138.030	84.770
138.890	83.560
132.740	77.140
136.553	81.823
3.330	4.101
1.923	2.368
138.640	84.270
141.990	87.080
138.170	83.160
139.600	84.837
2.083	2.021
1.203	1.167
